# High Spinal Cord Injury After a Minor Fall in a 23‐Month‐Old Girl With Atlantoaxial Instability Associated With Morquio Type A: A Case Report

**DOI:** 10.1155/cris/2693820

**Published:** 2026-07-23

**Authors:** Verneri Kryssi, Johanna Syvänen, Linda Helenius, Arimatias Raitio, Ilkka Helenius

**Affiliations:** ^1^ Department of Pediatric Surgery and Orthopedics, University of Turku and Turku University Hospital, Turku, Finland, utu.fi; ^2^ Department of Anesthesia and Intensive Care, University of Turku and Turku University Hospital, Turku, Finland, utu.fi; ^3^ Department of Orthopedics and Traumatology, University of Helsinki and Helsinki University Hospital, Helsinki, Finland, helsinki.fi

## Abstract

**Case:**

A 23‐month‐old girl with Morquio A syndrome (mucopolysaccharidosis IVA [MPS IVA]) scheduled for atlantoaxial stabilization surgery sustained a cervical spinal cord injury with tetraplegic symptoms following a low‐energy fall. Computed tomography (CT) images showed forward translation of C1 over C2 and magnetic resonance imaging (MRI) revealed upper cervical spinal cord injury. An urgent decompression and spinal fusion from C0 to C2 were performed. Postoperative recovery showed significant neurological improvement and at 1‐year follow‐up, the patient was symptom‐free.

**Conclusion:**

This case highlights the risk of severe cervical spinal cord injury following minor trauma in patients with MPS IVA‐associated cervical instability and demonstrates the potential for neurological recovery after prompt surgical decompression and stabilization.

## 1. Introduction

Morquio syndrome type A, also known as mucopolysaccharidosis IVA (MPS IVA; OMIM 253000), is an autosomal recessive lysosomal storage disorder [1, 2]. Its estimated prevalence is approximatelt 0.15–1.32 per 100,000 births [3–5]. The cause is defective N‐acetylgalactosamine‐6‐sulfatesulfatase (GALNS)‐enzyme, which results in progressive accumulation of glycosaminoglycans keratan sulfate (KS) and chondroitin‐6‐sulfate (C6S) in bone and cartilage, causing skeletal dysplasia, ligamentous hyperlaxity, and ossification disturbance [6–9]. The diagnosis is confirmed by a GALNS enzyme assay [9, 10].

Clinical manifestations generally begin before 1 year of age in severe phenotypes and during the second decade of life in milder forms. Affected infants typically appear normal at birth but develop progressive disease within the first few years of life, with over 70% exhibiting typical skeletal features by 2–3 years of age. Odontoid hypoplasia with atlantoaxial instability is among the most critical skeletal manifestations of MPS IVA. In combination with ligamentous laxity, incomplete ossification of the anterior and posterior rings of the atlas, and extradural glycosaminoglycan deposition, it can lead to atlantoaxial subluxation, cervical myelopathy, or even death [11, 12].

Neither enzyme replacement therapy with recombinant human GALNS enzyme nor hematopoietic stem cell therapy has been shown to have a significant effect on skeletal lesions [10]. Supportive measures remain the cornerstone of symptom management, while surgical intervention is often required as a preventive treatment or even as a life‐saving procedure. Due to the universal risk of atlantoaxial instability and spinal cord compression, regular screening is recommended and surgical stabilization should be considered when indicated [13].

We report a case of a 23‐month‐old girl with MPS IVA who sustained a high cervical spinal cord injury following a minor fall, necessitating emergency decompression and fusion. The aim of this case report is to highlight the risk of severe spinal cord injury associated with cervical instability and the role of urgent surgical decompression and stabilization in neurological recovery.

## 2. Case Report

A 16‐month‐old girl was referred to a pediatric orthopedic surgeon because of spinal curvature, rib flaring, and restricted shoulder joint movements. The patient was born full term, and no abnormalities were noted during routine child healthcare visits apart from mild hypotonia, later hypertonia, and shoulder joint stiffness. Brisk patellar and achilles tendon reflexes were observed, but otherwise, no neurological deficits were identified. Radiological examinations confirmed thoracolumbar scoliosis of 30°, mild thoracolumbar kyphosis, anterior remodeling of L2−4 lumbar vertebrae, and ovoid‐shaped thoracic vertebrae (Figure 1). Boston brace treatment was initiated for early‐onset scoliosis instead of serial body casting, considering the shape of the thorax and rib flaring. Whole‐spine magnetic resonance imaging (MRI) revealed upper cervical spinal stenosis at the atlantoaxial level, with a space available for the cord (SAC) of 5 mm (Figure 2) and a dysplastic odontoid process. Urinary glycosaminoglycan analysis demonstrated elevated levels of KS and dermatan sulfate, raising suspicion for MPS IV. Clinical exome sequencing identified variants in the GALNS gene (c.1055T >C, p.Leu352Pro and c.1156C >T, p. Arg386Cys), confirming the diagnosis of MPS IVA at 21 months of age.

**Figure 1:**
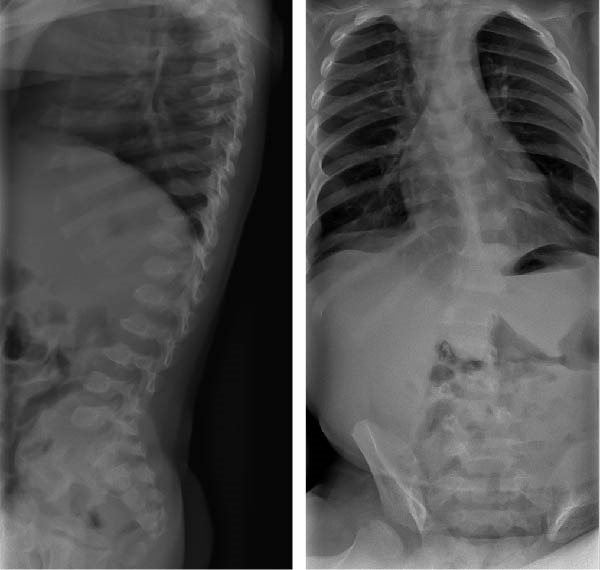
Spinal radiograph showing thoracolumbar scoliosis, ovoid thoracic vertebrae, and anterior hypoplasia of the lumbar vertebral bodies.

**Figure 2:**
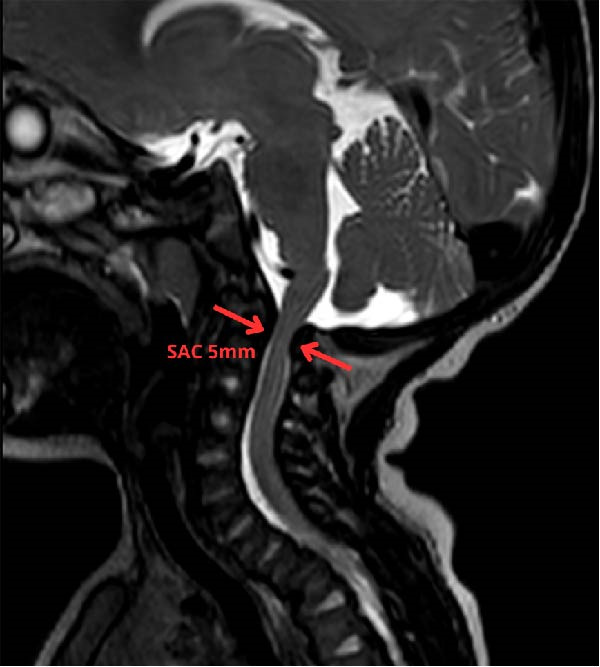
Pre‐traumatic sagittal T2‐weighted magnetic resonance image demonstrating severe atlantoaxial stenosis with complete effacement of the cerebrospinal fluid space surrounding the spinal cord. The space available for the cord (SAC) measured 5 mm and was determined as the shortest distance between the posterior margin of the anterior soft tissue/odontoid complex and the anterior surface of the posterior arch of C1 (red arrows).

Multidisciplinary preoperative evaluation involving a pediatric orthopedic surgeon, a pediatric spine surgeon, an anesthesiologist, a pediatrician, and a pediatric neurologist was deemed necessary before elective upper cervical decompression and stabilization. At 23 months of age, the patient was scheduled for elective decompression and prophylactic stabilization to address severe atlantoaxial stenosis and cervical spinal cord compression following evaluation by a pediatric spine surgeon. Dynamic flexion–extension radiographs and MRI were not considered necessary because of the markedly reduced SAC. Shortly thereafter, the patient fell approximately 40 cm from a sofa at home. Initially, she did not breathe spontaneously and quickly became cyanotic, and her mother began performing layperson cardiopulmonary resuscitation. After 5 min, she opened her eyes, and when paramedics arrived, oxygen saturation was 70%, rising to 98% with supplementary oxygen. In the emergency room, her oxygen saturation and hemodynamics were stable. However, the patient presented with tetraplegia, more pronounced in the left upper and lower extremities, and her head was slightly rotated to the right. Babinski reflexes were positive on both sides and muscle strengths were graded according to the International Standards for Neurological Classification of Spinal Cord Injury (ISNCSCI) as 1/5 in the left upper and lower extremities, 2/5 in the right upper extremity, and 3/5 in the right lower extremity.

Cervical computed tomography (CT) was performed in accordance with our trauma protocol, revealing forward translation of C1 over C2 with a 6 mm atlanto‐dental interval (ADI) and a high‐riding vertebral artery (HRVA) on the right side (Figure 3) with an isthmic height of 2,8 mm. Cervical T2‐weighted MRI demonstrated increased signal intensity of the spinal cord at C1–C2 level (Figure 4).

**Figure 3:**
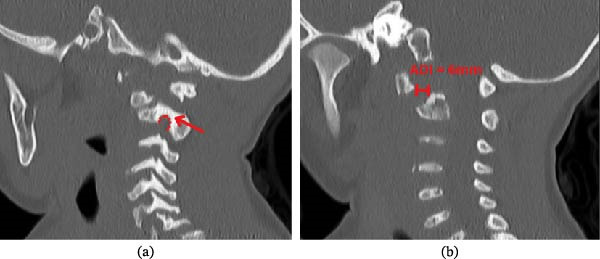
(a) A sagittal computed tomography image shows a narrowed isthmus of the right C2 pedicle (red arrow). Borders of the right transverse foramen of C2 are highlighted (red dots). The isthmus height was measured at 2.8 mm, which is indicative of a high‐riding vertebral artery. (b) A midsagittal computed tomography image illustrates anterior translation of C1 relative to C2. The red measurement marker indicates an atlanto‐dental interval (ADI) of 6 mm. Because the dysplastic cartilaginous odontoid process is not visible on CT, the ADI was measured from the posterior border of the anterior arch of C1 to the anterosuperior corner of the ossified body of C2. Slight rotation of the head was present because of the traumatic deformity.

**Figure 4:**
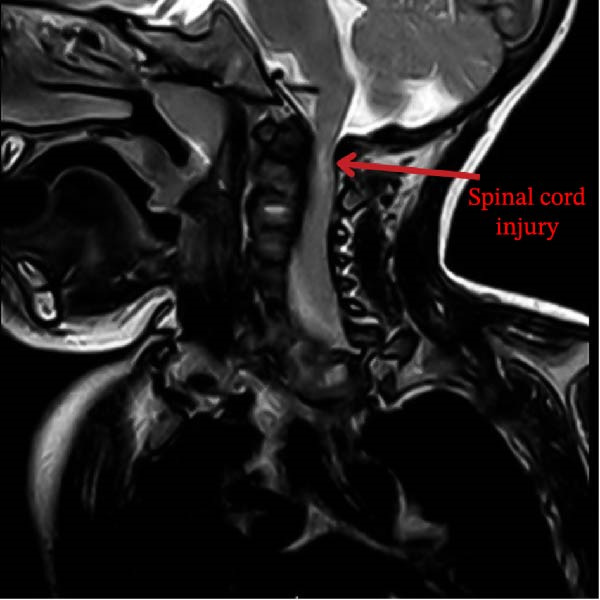
Post‐traumatic sagittal T2‐weighted magnetic resonance image illustrating increased intramedullary T2 signal intensity within the spinal cord at the C1–C2 level, consistent with spinal cord injury (red arrow). Severe atlantoaxial stenosis persists with complete effacement of the cerebrospinal fluid space around the cord. The space available for the cord (SAC) remained ~5 mm, similar to that observed on the pre‐traumatic MRI.

Urgent decompression and occipitocervical fusion from C0 to C2 were performed to stabilize the atlantoaxial segment and facilitate neurological recovery. Intraoperative neuromonitoring was not available during the procedure due to the emergency nature of the operation. Intubation was performed with manual in‐line stabilization of the head and neck with the aid of a nasal flexible bronchoscope. C1 laminectomy was performed and the nuchal ligament was excised. Spinal instrumentation with an occipital plate was used and C2 screws were inserted with a free‐hand technique (Vertex Max, Medtronic). A 3.5 mm pars screw was inserted into the left C2 pars, while a translaminar screw was placed on the right side because of the ipsilateral HRVA. Fixation of the occipital plate was performed with four 4.0 mm bi‐cortical screws (Figure 5). Pre‐contoured titanium alloy rods were introduced while pushing the C2 lamina downward to reduce the atlantoaxial instability. An autologous bone graft harvested from the left iliac wing was placed, and the correct implant position and reduction were verified using an O‐arm (Medtronic) spin (Figure 5). A cervical collar was applied, and the patient was transferred to the pediatric intensive care unit (PICU). A multidisciplinary rehabilitation program, including maintenance of an elevated mean arterial pressure and administration of intravenous dexamethasone, was initiated promptly, and gradual neurological recovery to full motor function was observed. At discharge 2 months later, the patient was able to walk independently and use both hands during play, with a preference for the right hand. At the 3‐month follow‐up, upper and lower extremity function was symmetric, although the patient’s mother reported noticing a slight weakness in the left hand. Cervical radiographs showed adequate implant positioning with an aligned atlantoaxial joint. At the 6‐month follow‐up, the patient’s neurological examination was normal except for brisk lower extremity reflexes. Cervical CT revealed bridging bone formation between the occiput and C2 (Figure 6), while MRI confirmed adequate decompression and reduced T2 signal intensity within the spinal cord at the site of the previously observed cord signal abnormality (Figure 7). Cervical collar treatment was discontinued. At the 12‐month follow‐up, the patient’s neurological status remained stable and no signs of implant‐related complications were observed.

**Figure 5:**
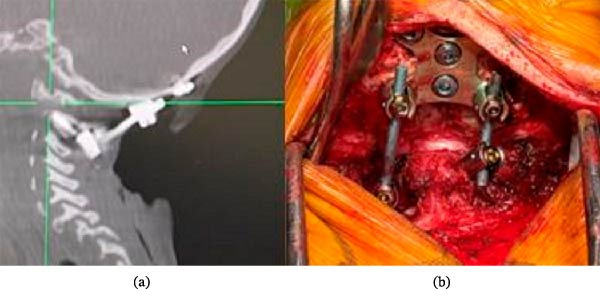
Sagittal projection with O‐arm confirming implant positioning (a) and photograph showing decompression and spinal instrumentation (b).

**Figure 6:**
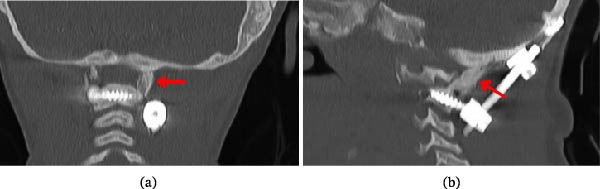
Computed tomography images show bridging bone formation at 6 months with coronal plane on (a) and sagittal plane on (b). Bridging bone formation is more extensive on the left side at the fixation level (red arrows).

**Figure 7:**
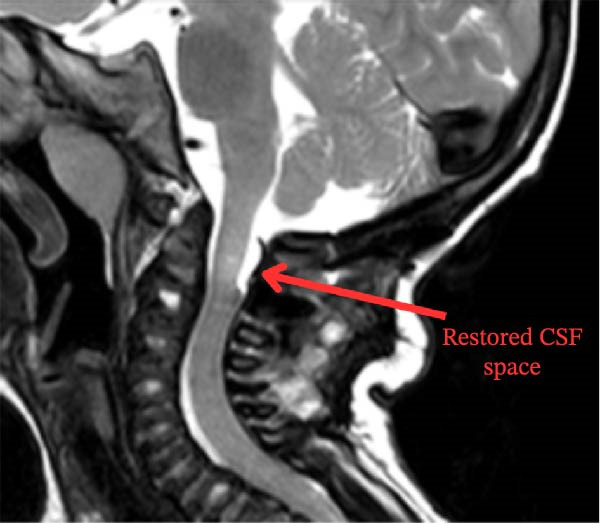
6‐month postoperative sagittal T2‐weighted magnetic resonance image demonstrating reduced signal intensity within the spinal cord and decompression at the level of C1−2, with restoration of the cerebrospinal fluid space surrounding the spinal cord (red arrow).

## 3. Discussion

Patients with MPS IVA often require major surgical procedures involving the cervical spine, hips and knees, and orthopedic complications are among the most significant clinical concerns. Early diagnosis is essential for improving the quality of life and enabling timely treatment of orthopedic manifestations [8, 11]. Atlantoaxial instability in patients with MPS IVA is a progressive disorder resulting usually from odontoid hypoplasia, ligamentous laxity, and glycosaminoglycan deposition around the craniovertebral junction. Increasing instability and soft tissue thickening may lead to cervical canal stenosis and spinal cord compression. Although some patients remain asymptomatic for prolonged periods, neurological deterioration may occur gradually or be precipitated by minor trauma. Regular clinical and radiological surveillance is essential to identify patients at risk for neurological injury and to guide the timing of surgical stabilization [13, 14].

No universally accepted guidelines for prophylactic atlantoaxial stabilization are so far available for MPS IVA patients, nor is there a defined minimum SAC threshold indicating surgery. Assessment of the SAC in patients with MPS IVA is often complex because glycosaminoglycan accumulation within the peri‐odontoid soft tissue contribute to spinal canal narrowing. Consensus recommendations from the World Federation of Neurosurgical Societies (WFNS) suggest that surgical decompression and stabilization should be considered even in asymptomatic patients with spinal cord compression or atlantoaxial instability. Surgical intervention should also be considered in patients with signs and symptoms of myelopathy, significant instability with SAC <12 mm or spinal cord signal change on T2‐weighted MRI [13–16].

This case also raises the question of prophylactic stabilization in patients with significant atlantoaxial instability. Although the optimal timing of surgery remains individualized, the present case illustrates the potentially catastrophic neurological consequences of untreated instability. Severe cervical instability is associated with substantial morbidity and premature mortality if left untreated. Even a minor trauma, such as a fall, may result in a spinal cord injury, as demonstrated in our patient. Therefore, cervical compression should be managed proactively when significant stenosis or instability is identified [8].

In our patient, pre‐traumatic MRI revealed critical cervical stenosis with complete effacement of the cerebrospinal fluid space surrounding the spinal cord and a diminished SAC of 5 mm. Despite the absence of neurological deficits before the injury, these findings indicated advanced canal compromise and supported the need for surgical stabilization. Following the fall, CT exhibited an increased ADI, confirming atlantoaxial instability [10, 13, 14].

In young children, small and fragile bony elements restrict the surgical fixation points [17]. Atlantoaxial instability is usually addressed with C1–C2 instrumented fusion, when technically feasible, and rigid instrumentation using screws and rods improves fusion rates [13, 17]. Although C1 lateral mass fixation is an option, it may be difficult to perform in patients with skeletal dysplasia because of abnormal anatomy and limited osseous dimensions [17, 18]. While isolated C1–C2 fusion is generally preferred because it preserves occipitocervical motion, this option was not considered feasible in our patient. Marked C1 dysplasia and small osseous dimensions limited the available fixation points and increased the risk of neurovascular injury. Furthermore, the procedure was performed on an emergency basis following acute spinal cord injury, necessitating rapid and reliable stabilization. Therefore, occipital fixation was incorporated into the construct to achieve stable fixation while minimizing the risk of iatrogenic complications. C2 pedicle screws typically provide reliable fixation but carry a risk of VA injury [18, 19]. Post‐traumatic CT demonstrated a diminished right C2 pedicle height consistent with a type 1 HRVA according to the Klepinowski classification [20, 21]. Because translaminar screw fixation minimizes the risk of intraoperative VA injury, is technically simpler than pedicle screw insertion, and offers biomechanically efficient fixation with high fusion rates, a translaminar screw was used on the right side of C2 [22].

To ensure successful fusion, generous autografting is recommended in patients with MPS IVA due to impaired bone formation [10, 13, 19, 23]. The amount of autograft bone available for grafting in young children is limited. The iliac crest is often the optimal site for harvesting a sufficient volume of bone and is associated with higher fusion rates than occipital bone grafts or allograft bone [24, 25]. Bony fusion after atlantoaxial fixation can take up to 6 months [19]. Postoperatively, a halo vest or rigid cervical collar is typically used for immobilization. In our patient, a rigid collar was maintained for 6 months, and fusion was confirmed by CT. Both short‐term and long‐term fusion rates after posterior cervical fusion have been shown to be favorable in patients with MPS IVA. Interestingly, following upper cervical fusion, the odontoid process appears to ossify normally. Long‐term orthopedic follow‐up is essential to detect and manage subaxial instability, scoliosis, and lower extremity disorders [8, 13, 19, 25, 26].

In patients with Morquio syndrome older than 5 years of age, annual neurological examinations and spinal imaging are recommended [25]. After upper cervical fusion, distal junction instability has been reported during long‐term follow‐up, and spinal cord compression may subsequently develop in a substantial proportion of patients [26]. Patients who have neurological impairment prior to upper cervical fusion tend to continue deteriorating after surgical intervention, whereas asymptomatic patients generally achieve better long‐term outcomes following fusion [27].

## 4. Conclusion

This case presented a challenging emergency upper cervical fusion and decompression in a young MPS IVA patient with severe neurologic injury after a minor fall. Although neurological impairments were fully resolved and fusion was achieved, the progressive nature of the disease makes additional orthopedic interventions likely, necessitating long‐term regular follow‐up and coordinated care from a multidisciplinary team.

## Author Contributions

**Verneri Kryssi:** data curation, visualization, writing – original draft, writing – review and editing. **Johanna Syvänen**: conceptualization, investigation (surgery performed), writing – review and editing. **Linda Helenius:** investigation (performed anesthesia), writing – review and editing. **Arimatias Raitio:** project administration, writing – review and editing. **Ilkka Helenius:** conceptualization, data curation, investigation (surgery performed), project administration, visualization, writing – review and editing.

## Funding

The authors declare that no funding or financial support was received for this study. Open access publishing facilitated by Turun yliopisto, as part of the Wiley ‐ FinELib agreement.

## Disclosure

Investigation performed at Turku University Hospital, Turku, Finland.

## Ethics Statement

Written informed consent was obtained from the patient’s legal guardians for publication of this case report and any accompanying images. No ethical approval was required for this case report in accordance with the institutional guidelines.

## Conflicts of Interest

The authors declare no conflicts of interest.

## Data Availability

Data sharing is not applicable to this article as no datasets were generated or analyzed during the present study.
